# Methicillin-resistant *Staphylococcus aureus* and pulmonary outcome in people with cystic fibrosis: a European Cystic Fibrosis Patient Registry data analysis

**DOI:** 10.1183/23120541.01284-2024

**Published:** 2025-09-01

**Authors:** Meir Mei-Zahav, Miri Dotan, Luigi Annicchiarico, Annalisa Orenti, Dario Prais

**Affiliations:** 1 Kathy and Lee Graub Cystic Fibrosis Center, Schneider Children's Medical Center of Israel, Petach Tikva, Israel; 2 School of Medicine, Faculty of Medical and Health Sciences, Tel Aviv University, Tel Aviv, Israel; 3 Department of Clinical Sciences and Community Health, Dipartimento di Eccellenza 2023-2027, Laboratory of Medical Statistics, Biometry and Epidemiology “G.A. Maccacaro”, University of Milan, Milan, Italy; 4 These authors contributed equally

## Abstract

*Staphylococcus aureus* is an early and common airway infection in people with cystic fibrosis (pwCF), peaking in adolescence and declining in adulthood [1]. Most infections are methicillin-sensitive but methicillin-resistant *Staphylococcus aureus* (MRSA) prevalence has risen in North America, from 2.1% in 1996 to 18.0% in 2021 [2]. While some studies link MRSA to severe lung infections, lower lung function and higher mortality in pwCF, its association with clinical outcomes remains unclear, with some studies showing effects mainly in children [3–5]. Using the European Cystic Fibrosis Society Patient Registry (ECFSPR), we aimed to assess MRSA prevalence and its association with demographic and clinical factors, comparing adult and paediatric populations.

## Shareable abstract

**MRSA in CF patients is linked to worse lung function, longer *i.v.* antibiotic treatments, increased hospital stays and higher risks of complications. This underscores the importance of continuous MRSA monitoring and targeted interventions in CF care.** https://bit.ly/43Gjjpt

*To the Editor:*

*Staphylococcus aureus* is an early and common airway infection in people with cystic fibrosis (pwCF), peaking in adolescence and declining in adulthood [1]. Most infections are methicillin-sensitive but methicillin-resistant *Staphylococcus aureus* (MRSA) prevalence has risen in North America, from 2.1% in 1996 to 18.0% in 2021 [2]. While some studies link MRSA to severe lung infections, lower lung function and higher mortality in pwCF, its association with clinical outcomes remains unclear, with some studies showing effects mainly in children [3–5]. Using the European Cystic Fibrosis Society Patient Registry (ECFSPR), we aimed to assess MRSA prevalence and its association with demographic and clinical factors, comparing adult and paediatric populations.

In this cross-sectional analysis of the data gathered by the ECFSPR in 2019 from 38 European countries [6], we examined MRSA prevalence and its associations with demographics and clinical factors. We opted for 2019 as it served as a valuable pre-pandemic and pre-widespread adoption of highly effective transmembrane conductance regulator (CFTR) modulators reference point. MRSA status was based on respiratory cultures reported to the ECFSPR, and we assessed MRSA prevalence by country, income as defined by Gross National Income (GNI), age, comorbidities, therapies and respiratory infections. Low income was defined as a GNI *per capita* (computed using Atlas methodology) lower than USD 20 000. Chronic infection was determined at the time of data entry using modified Leeds criteria.

Prevalence of MRSA was computed separately according to country, age group and genotype group. The clinical characteristics of pwCF were described after stratifying pwCF according to MRSA infection status. Simple regression models were fitted to investigate the association between MRSA infection and each clinical characteristic.

Furthermore, multiple linear regression models were used for numerical outcomes: forced expiratory volume in 1 s (FEV_1_) % of predicted, number of days on intravenous antibiotics and number of days in the hospital. Multiple logistic regression models were used for dichotomous outcomes: pneumothorax, haemoptysis, transplantation or death. The multiple regression models were adjusted for age, gender, age at diagnosis, genotype, body mass index (BMI), diabetes, *Pseudomonas aeruginosa* infection, *Burkholderia cepacia* complex infection, CFTR modulator use and socioeconomic status.

This study was approved by the ECFSPR Scientific Committee and by each member of the ECFSPR Steering Group, which consisted of representatives from the participating countries. The pwCF provided informed consent to be included in the ECFSPR.

Of the 48 099 pwCF included in the study (24 295 under the age of 18 years and 23 804 adults), MRSA was reported in 2494 pwCF. The prevalence was 5.19% (95% CI 4.99–5.39%) and varied significantly by country of residence; higher prevalence was found in the south and east of Europe, and in low- compared to high-income countries (6.99% *versus* 4.78%, p<0.001). MRSA prevalence increased with age, reaching two peaks at 16–19 years (6.6%) and at 30–34 years (6.7%); however, it did not differ significantly by gender. MRSA was more prevalent among patients with a minimal function genotype (mutation type I, II or III) than those with a residual function genotype (mutation type IV or V) (5.59% *versus* 3.55%, p<0.001).

When comparing MRSA negative and positive pwCF, it emerged that MRSA infection was associated with treatment with dornase alfa (58.8% *versus* 69.4%, p<0.001), inhaled antibiotics (42.7% *versus* 52.0%, p<0.001), macrolides (29.6% *versus* 35.6%, p<0.001), inhaled steroids (30.2% *versus* 40.0%, p<0.001) and CFTR modulators (18.9% *versus* 22.1%, p<0.001).

Compared to those without chronic MRSA, clinical status was worse among those with chronic MRSA, as defined by lower FEV_1_ % of predicted across all age groups (figure 1) and lower body mass index z-score (−0.23 *versus* −0.38, p<0.001). Among those with chronic MRSA infection, the prevalence of lung transplants was lower (5.28% *versus* 2.53%, p<0.001), but the proportions of death were similar (0.62% *versus* 0.68%, p=0.7). The association between MRSA and lung transplant remains evident even after stratifying by income status. Figure 1 shows a regression model of FEV_1_ % of predicted trends by MRSA status. FEV_1_ % of predicted was significantly lower in pwCF with chronic MRSA infection aged 6 (p=0.025) to 50 years (p=0.012). When this was stratified by country, in low-income countries, differences were largely insignificant (except at age 39 years). In high-income countries, individuals with chronic MRSA infection had significantly lower FEV_1_ % of predicted across all ages.

**FIGURE 1:**
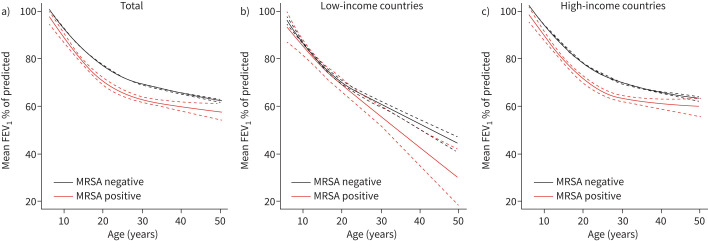
The trend of forced expiratory volume in 1 s (FEV_1_) % predicted at different ages according to methicillin-resistant *Staphylococcus aureus* (MRSA) status. a) Total, b) low-income countries and c) high-income countries.

In the univariable models, MRSA was found to be more prevalent in the presence of chronic *P. aeruginosa*, MRSA, *Stenotrophomonas maltophilia* and *Achromobacter*: odds ratios 1.35, 1.47, 1.5 and 1.6, respectively; p<0.001 for all. Co-infection rates remained consistent when analyses were restricted to pwCF with class I–III genotypes, showing minimal deviation from the estimates of the entire cohort.

Among pwCF who were positive for MRSA, compared to those who were negative, FEV_1_ % of predicted was 4.94% lower, intravenous antibiotic use was longer by a median of 6.14 days, and hospitalisation was a median of 4.34 days longer. Patients who tested positive for MRSA compared to those who did not were nearly twice as prone to experience incidents of haemoptysis or pneumothorax (OR 1.90, 95% CI 1.4–2.4). The proportion of death did not differ significantly between the groups.

When we considered the adult and paediatric populations separately, all the parameters mentioned above, including genotype, pancreatic insufficiency, BMI, FEV_1_ % of predicted, microbiology, treatments and complications, remained significantly different between the MRSA-positive and -negative groups in both the adult and the paediatric population (data not shown).

The 2019 ECFSPR data reported a 5.2% MRSA prevalence among 48 099 pwCF. Notably, MRSA was more common in lower-income European countries, possibly due to lower infection control adherence and higher lung disease severity in these regions [7]. The annual epidemiological report for 2019 on antimicrobial resistance in the European Union highlighted significant inter-country disparities in MRSA prevalence. Generally, southern and eastern European regions exhibited higher resistance percentages than northern Europe. Thus, background geographic variability in MRSA prevalence can be the leading cause for higher MRSA prevalence in the cystic fibrosis population [8]. Further supporting this point is the significantly higher prevalence of MRSA among pwCF in North America – 18% according to a patient registry report [2] – which represents the higher background MRSA prevalence in that region [9]. The observed geographic variability in MRSA prevalence among pwCF could be influenced by differences in healthcare infrastructure, access to advanced cystic fibrosis care and antibiotic stewardship programmes across countries. Genetic predispositions and environmental factors unique to certain populations could contribute to the variability in MRSA prevalence and outcomes.

MRSA-positive pwCF showed worse clinical status, including lower FEV_1_ % of predicted and BMI, increased hospitalisations, and cystic fibrosis complications like haemoptysis. Lung transplantation was less common among MRSA-positive patients, perhaps because MRSA is considered a relative contraindication for lung transplantation in some centres [10]. Another explanation is that MRSA is more prevalent in low-income countries, where lung transplant rates are lower. However, as stated in our results, the association between MRSA and lung transplant remains significant even after adjusting for income status.

Similar to our findings, in a cohort of 20 451 individuals from the North American cystic fibrosis registry, MRSA-positive patients had lower FEV_1_ % of predicted and higher proportions of hospitalisation and antibiotic use [11]. In another North American study, FEV_1_ % of predicted declined more rapidly among those with persistent MRSA [12]. Determining whether MRSA is the underlying cause or merely an indicator of more severe disease due to a combination of risk factors remains challenging and requires further clarification in a long-term prospective study. MRSA colonisation could be a proxy indicator of higher healthcare utilisation rather than a direct cause of worsened clinical outcomes.

We found that MRSA prevalence was notably higher in the presence of chronic *Pseudomonas aeruginosa, S. maltophilia* and *Achromobacter*. Colonisation with these bacteria might be associated with increased use of antibiotics and frequent hospital visits. An association between MRSA and *Achromobacter* infections was previously documented [13]. Prior epidemiological studies from the US patient registry identified *P. aeruginosa* infection as a risk factor for both the acquisition of MRSA [12] and its persistence, compared to clearance, following an initial infection [14]. These co-infections could collectively contribute to worse clinical outcomes, with MRSA being one piece of a complex microbial interplay.

Effective MRSA management is challenging due to antibiotic resistance, biofilm formation and anaerobic growth, with no current cystic fibrosis-specific treatment guidelines [3]. Despite the limitations of registry cross-sectional data, this study underscores the need for continued MRSA surveillance, infection control and targeted interventions in cystic fibrosis care. Further research should focus on prevention and management strategies for MRSA in the cystic fibrosis population.
